# Environmental Attitudes, Behaviors, and Responsibility Perceptions Among Norwegian Youth: Associations With Positive Youth Development Indicators

**DOI:** 10.3389/fpsyg.2022.844324

**Published:** 2022-02-23

**Authors:** Maria Bøhlerengen, Nora Wiium

**Affiliations:** ^1^Department of Education, Inland Norway University of Applied Sciences, Oppland, Norway; ^2^Department of Psychosocial Science, University of Bergen, Bergen, Norway

**Keywords:** positive youth development, environmental attitudes, conservation behavior and intention, environmental responsibility, Norway

## Abstract

Young people’s environmental attitudes and behaviors are essential for environmental conservation, hence the need to identify facilitating factors. Promoting positive development among young people may empower them to contribute actively to their environment through positive attitudes and behaviors. In the present study, we examine the associations between the 5Cs of Positive Youth Development (Character, Confidence, Connection, Caring, and Competence) and environmental concerns among Norwegian youth, measured through environmental attitudes, conservation behavior, and responsibility. Cross-sectional data on demographic variables, the 5Cs and environmental concerns were collected from high school students (*N* = 220, *M*_*age*_ = 17.30, *SD* = 1.12). For results, Character was associated with several of the environmental variables (β*s* = 0.28–0.58, *p* < 0.05), followed by Competence (β*s* = 0.26–0.31, *p* < 0.05) and Caring (β*s* = 0.23, *p* < 0.05), and finally Confidence, which showed a negative association with conservation behavior (β = −0.29, *p* < 0.05). There was no significant association with Connection. While these preliminary findings pave the way for future research that should involve more representative samples, the significant associations between several of the 5Cs and the environmental factors may have some implications for policy and programs on youth development and sustainable behaviors.

## Introduction

Since the mid-1980s, scientists and the media have tried to communicate the dangers of man-made climate change to the public (Moser, 2010). Temperature changes because of global warming have led to the melting of snow and ice, elevated sea levels as well as frequent and extreme weathers, among others (Environment Directorate (Miljødirektoratet), 2018; Intergovernmental Panel on Climate Change, 2018; World Meteorological Organization, 2021). In April 2021, at the lunch of the World Meteorological Organization’s report on the State of the Global Climate 2020, the United Nation’s Secretary General declared 2021 as “the make it or break it year,” referring to the report’s emphasis on accelerating climate change indicators and the aggravating impact. To limit the damage caused by these changes and to achieve sustainable development, a complete reversal of environment-distractive actions is needed in the next years. Equally important for future environmental protection is today’s youth’s knowledge, attitudes, and behaviors toward the environment (Wray-Lake et al., 2010). In line with the Positive Youth Development (PYD) framework (Lerner et al., 2015), young people can contribute to a better future if they are equipped with the skills and resources needed for active community participation. The present study seeks to explore the environmental concerns and contribution of youth living in Norway within the PYD framework.

### The Climate Fight, Youth Actions, and the Norwegian Context

Since the industrial revolution in the mid-19th century, human activity has increased the content of greenhouse gases in the earth’s atmosphere, and consequently, the average temperature on the earth’s surface (Melillo et al., 2014; World Meteorological Organization, 2021). To date, the temperature has increased by about one degree centigrade (i.e., 1.2°C) compared to pre-industrial times. Negative consequences, such as droughts, floods, and storms, will not only affect people’s living conditions, but their health as well. Given the serious consequences that changes in the climate and environment can create, it is important that young people can contribute to climate and environmental issues as these will affect their future opportunities and wellbeing. Today, there are young people who try to take control of their own lives, health, and future by focusing on the climate and environment. An example is the Swedish Greta Thunberg and her climate strike initiative. The initiative started in 2018 with 15-year-old Thunberg sitting outside the parliament in Sweden every Friday and fighting for better actions related to climate change (TedX Talks, 2018). In 2019, several school strikes for climate in major cities around the world were staged. During the recent 2021 climate change summit in Glasgow, United Kingdom, young people actively participated either virtually or in-person to demand concrete and better actions from the world leaders.

In Norway, climate change strikes have been arranged across the country (Norsk Rikskringkasting AS [NRK], 2019), with Norwegian youth contributing also to the focus on climate and environmental problems facing the world. Norway is a constitutional monarchy and a parliamentary democracy (Thuesen et al., 2019). In parliamentary elections, all Norwegian citizens above the age of 18 who are enlisted in the population register as residents in Norway, have voting rights (in line with the Norwegian Constitution that was adopted in 1814). This ensures the influence of adult Norwegian citizens in democracy to promote the political issues they perceive as important, such as climate and the environment. As Norwegian youth do not have the right to vote until they turn 18 (as is the case for most of our study participants), it is interesting to examine the attitudes they have concerning who they think is responsible for the climate and environment. Do they see it as their personal, the consumer’s or the government’s responsibility to influence the climate and environment?

One of the largest target groups for the climate movement within politics in Norway is young Norwegians (Sørenes, 2019). In January 2021, about 23% of the total Norwegian population of 5,391,369 was under the age of 20 (Statistics Norway, 2021). This is not a particularly large percentage, but political parties with climate as their main concern, focus on qualified youth as their target group. Among other things, the Green party (MDG) during the election campaign before the municipal and county elections in 2019, focused especially on young voters, to engage them in using their right to vote for the climate and environment (Sørenes, 2019). Political parties in Norway are not the only ones who see the importance of youth’s commitment to the climate and environment. Several organizations that focus on climate and the environment do the same. Among these are Nature and Youth, and the Future in Our Hands. Nature and Youth have over 8000 members divided into 70 local teams (Nature and Youth, 2019). The organization works to find solutions to environmental problems and take up the fight to stop companies that are “destroying” the environment. The Future in Our Hands has about 30,000 members and has reduction in consumption of natural resources, global redistribution, and business ethics (e.g., monitoring ethical standards on Norwegian foreign investments) as their main goals (The Future in Our Hands, 2019). These organizations give Norwegian youth the opportunity to contribute to local teams, boards and voluntary work for the climate and environment. Indictors of positive development that may influence Norwegian youth contribution to the environment have yet to be investigated, a topic we concentrate on in the present study.

### Positive Youth Development and Environmental Concerns

Positive Youth Development emerged as a theoretical perspective in the 1990s as an alternative to the earlier deficit approach on youth research. This new way of addressing young people emphasized that all young people have strengths that can be harnessed (Lerner et al., 2005, 2018). PYD with its foundation in relational developmental systems theory, underscores the importance of plasticity in youth development (i.e., the possibility of systematic change in human development; Overton, 2003). The potential for plasticity exists because of a mutually influential and bidirectional relationship between the individual and their context, which includes the family, school, and community (Lerner et al., 2005). The skills, resources and opportunities embedded in the dynamic individual-context relationship are expected to facilitate positive development.

The 5Cs: Character, Confidence, Connection, Caring, and Competence (Roth and Brooks-Gunn, 2003; Geldhof et al., 2014) are often used as indicators of positive development within the PYD theoretical framework. Character reflects among other things, respect for the rules of culture and society, standards of correct behavior and the presence of an inner moral compass. Confidence represents an inner experience of overall positive self-esteem and self-image. Connection is the healthy ties to people and institutions that are reflected in mutual contributions in the relationship. Caring signifies the experience of sympathy and empathy for others, while Competence indicates a positive view of one’s actions in domain-specific areas, such as social, academic, and vocational (Roth and Brooks-Gunn, 2003; Geldhof et al., 2014). Consistent with Lerner (2004), the presence of the 5Cs can lead to a sixth C, namely, Contribution. Young people engage in behaviors that indicate the sixth C when they make a positive contribution to the self, family, and community. Accordingly, by promoting the 5Cs, young people can be empowered to contribute to their contexts, including the climate and environment.

Using data from “Monitoring the Future Study” (Johnston et al., 2006), Wray-Lake et al. (2010) examined the opinions, attitudes and behaviors related to climate and environmental change among high school students in the United States, from 1976 to 2005. Based on the different waves of data collected, the authors found that the students were more likely to place greater emphasis on government and consumer’s responsibilities for the environment than on their own personal responsibility. Thus, these students did not see themselves as instrumental in the fight against climate change. Nevertheless, Wray-Lake et al. (2010) suggest that it is important to listen to youth’s ideas and inclinations when it comes to creating a sustainable future. Reiterating the PYD framework, one way to support young people in the fight for a sustainable future will be to support their positive development. This can provide them with the opportunity to be active community members with the power to influence the future.

Research investigating the associations between the 5Cs and environmental concerns is limited and even so in the Norwegian context. In one study among 995 Ghanaian youth, Kabir and Wiium (2021) found a positive association between Character and a sense of consumer and government environmental responsibility. Confidence and Caring were positively associated with attitudes toward pollution, although Confidence was also negatively related to environmental conservation intentions. Furthermore, Competence was negatively related to attitudes toward pollution while Connection was not significantly related to any of the environmental factors. Thus, the findings perhaps suggest the relative importance of the 5Cs to environmental factors. In another study involving a geographically and racially diverse sample of 2467 American youth, Metzger et al. (2018) found among others that empathy predicted environmental behavior (e.g., turning off electronics when not in use). Metzger et al. (2018) finding appears to support a possible link between Caring (which assesses empathy and sympathy for others) and youth engagement in environmental behaviors.

A distal but relevant study carried out among adults in Netherlands by van der Werff et al. (2013) found that it was possible to act environmentally friendly without external incentives. The study investigated the relationship between intrinsic values and environmentally friendly behavior by examining whether participants in the study were intrinsically motivated to act sustainably and environmentally. The authors argued that some people act environmentally friendly even though it could be cumbersome and without external incentives. van der Werff et al. (2013) believe that motivation to act environmentally friendly can be explained as commitment-based inner motivation, and compares this type of motivation to personal norm, an attribute that by definition is closely related to Character, one of the 5Cs of PYD.

Like van der Werff et al. (2013), Balunde et al. (2020) observed the importance of personal norms to various pro-environmental behaviors in a series of studies among adolescents in Lithuania, and found in one study, associations with general behaviors, such as recycling, environmentally friendly traveling and purchasing environmentally friendly goods. In another study involving more specific pro-environmental behaviors, biospheric values reflecting the adolescents’ general environmental considerations, were indirectly related to three pro-environmental behaviors (i.e., recycling non-refundable plastic, cycling to school and purchasing organic food products) *via* environmental self-identity and personal norms. Thus, the authors found that environmental consideration was related to both general and specific pro-environmental behaviors. In another study involving both youth and adults in Germany, Misch et al. (2021) found a positive link between moral identity (i.e., how being moral is central to personal identity) and support of the youth climate movement Fridays for Future (F4F), a movement of students that skip school on Fridays to protest for better climate policy.

These earlier studies help to lay the foundation for the idea that environmentally friendly attitudes and behaviors can be influenced, and that the sense of responsibility for the environment can be part of the inner motivation that leads to youth contribution to the environment. Thus, while research on youth’s contribution to the environment within the PYD framework is limited, the above-mentioned studies on how environmentally friendly actions can be intrinsically motivated could suggest that environmental attitudes, behaviors, and responsibility perceptions among Norwegian youth can be facilitated by their experience of positive indicators, such as the 5Cs of PYD.

### Aims of the Present Study

This manuscript involves young people in Norway, their environmental attitudes, behaviors and responsibility perceptions, and associations with the 5Cs. Many of the challenges of communication about the environment and climate are that the consequences are long-term, and it can therefore be difficult to perceive the situation as urgent (Moser, 2010). However, the long-term scope means that young people are given a key role in fighting for climate and environmental change. Besides, identity formed during adolescence can predict attitudes, values, and behaviors throughout life (Wray-Lake et al., 2010). Against this background, young people’s attitudes toward climate and the environment can predict future climate policy and societal attitudes to climate and environment change. Based on the PYD theoretical assumption concerning the positive relationship between the 5Cs and youth contribution, alongside the reviewed limited research, we hypothesize that a greater experience of the 5Cs will be associated with higher scores on the environmental factors.

By examining whether there is a positive association between Norwegian youth’s experience of the 5Cs and their attitudes, behaviors and responsibility perception for the environment, the present study seeks to shed light on how one can promote youth participation and contribution to the environment. If there is a link between the 5Cs and the environmental factors, the PYD framework will be deemed as suitable to inform further research, policy, and program on youth participation in sustainable development.

## Materials and Methods

### Participants

A cross-sectional study was conducted among students from four high schools in Eastern and Western Norway, which were selected through convenience sampling. The present study forms part of a larger international project on positive youth development that seeks to examine the role of youth strengths (e.g., social competence) and contextual resources (e.g., support at school or home) in youth development (Wiium and Dimitrova, 2019). A total of 220 students (52% males) from the four schools participated in the survey, with an age range of 16 to 20 years (*M*_*age*_ = 17.30, *SD* = 1.12). About 83 and 87% reported that the highest level of their father’s and mother’s education, respectively, was post-secondary.

### Measures

An online questionnaire containing items on the 5Cs, environmental concerns and demographics was created for data collection.

#### The 5Cs of Positive Youth Development

The short form of the PYD scale containing 34 items (Geldhof et al., 2014) was used. Sample items for the 5Cs: Character, Confidence, Connection, Caring, and Competence were, “I usually act the way I am supposed to,” “I really like the way I look,” “I am a useful and important member of my family,” “It bothers me when bad things happen to any person,” and “I have a lot of friends,” respectively. Responses ranged on a 5-point Likert scale, for example, from 1 (Strongly disagree) to 5 (Strongly agree), with higher scores indicating more of one of the Cs. Cronbach’s alphas of the 5Cs were 0.93 (Character), 0.86 (Confidence), 0.89 (Connection), 0.85 (Caring), and 0.88 (Competence).

#### The Environmental Variables

To study environmental concerns reflecting attitudes toward increased pollution, environmental conservation behavior and intention, and environmental responsibility, a list of items from Wray-Lake et al. (2010) was adopted. Attitudes toward pollution was assessed using three items that dealt with increase in pollution, the dangers involved, and the significance of pollution relative to growth. Responses were on a 5-point Likert scale, ranging from 1 (Disagree) to 5 (Agree), with higher scores indicating stronger positive attitudes to safeguard the environment and reduce pollution. Cronbach’s alpha of the three items was only 0.41 and as such, the items were addressed separately in data analysis.

Environmental conservation behavior and intention (Wray-Lake et al., 2010) were measured using four items. Three items examined positive conservation behavior and one examined conservation intention. Items measuring conservation behavior were related to reducing heat in winter in order to save electricity, cutting down on driving in order to save fuel and reducing the amount of electricity used in order to save energy. Responses were rated from 1 (Not at all) to 4 (Yes, quite a lot). The measurement of conservation intention was related to the willingness to use a bike or mass transit (if available) rather than a car to get to work. Responses were rated on a 5-point Likert scale from 1 (Disagree) to 5 (Agree). Cronbach’s alpha of the three items that measured conservation behavior was 0.81, showing very high reliability. A mean score of the three items was created for further analyses. The item measuring conservation intention was addressed separately, and not together with behavior, based on findings that intention does not always correlate with behavior (Sheeran and Webb, 2016).

Environmental responsibility was measured using five items from Wray-Lake et al. (2010). One item examined personal responsibility, another examined consumer responsibility and the remaining three items examined government responsibility. The items related to personal and consumer responsibilities reflected how much effort participants made to conserve energy and protect the environment concerning the things they buy or do, and how much they think people will have to change their buying habits and way of life to correct environmental problems, respectively. The first item that assessed government responsibility was about how the state should take action to solve environmental problems even if the use of certain products would have to be changed or banned. The second concerned the placing of higher taxes on products that cause pollution in their manufacture or disposal, so that companies can find better ways to produce them, while the third was about the steps that the state needed to take to deal with environmental problems, even though it may involve higher prices or taxes. Responses for all three responsibility types were on a 5-point Likert scale, ranging from 1 (Disagree) to 5 (Agree). The three questions measuring government responsibility had a Cronbach’s alpha of 0.90 (indicating high reliability) and were treated together as a mean score in the data analysis.

#### Demographic Variables

In addition to the 5Cs and environmental variables, demographic data that were collected and included in the present study were age, gender (male or female) as well as father and mother’s highest completed education [five levels of education: 1 (no education), 2 (primary school), 3 (high school), 4 (technical or vocational school), and 5 (university)]. These data were treated as control variables to ensure that they did not confound the role of the 5Cs in the environmental factors.

### Procedure

Prior to data collection, Semantix Translations Norway AS, a company that specializes in interpretation and translation services, translated the questionnaire from English to Norwegian, using a double-checking procedure and experts in the related field of research to ensure that the meaning is preserved. Headmasters of the selected high schools were contacted *via* e-mail with a request to participate in the study and an information letter about the purpose of the study. After agreeing to participate, schools were sent an informed consent form, which they were asked to sign and then send back. Once that was done, five teachers from the four schools that agreed to conduct the survey with their students were contacted *via* email with a link to the electronic questionnaire. Before the students could complete the survey, they read and signed an informed consent developed for participants. The survey, which was approved by NSD–Norwegian Centre for Research Data (51708/3/IJJ), was conducted in May–August 2019, and it took approximately 30 min for participants to complete the questionnaire.

### Data Analysis

Missing values on study variables ranged between 0% on gender and 13% on questions regarding the 5Cs and were handled with pairwise deletion, a procedure that excludes cases from the analysis when data is missing, and vice versa. In preliminary analyses, the linearity and normal distribution of the environmental factors as dependent variables were determined, with skewness and kurtosis falling within the acceptable range of −2 to +2 and −7 to +7, respectively (Byrne, 2010). Reliability analyses were run on the various scales measuring each of the 5Cs, attitudes toward pollution, conservation behavior and responsibility perception for the environment. Mean scores were created for the scales (i.e., the 5Cs, conservation behavior and government responsibility) that showed good internal reliability. Then, to assess the patterns among the demographic variables, the 5Cs and environmental variables, frequency and descriptive analyses were performed (Table 1).

**TABLE 1:** Study variables among high school students in Norway.

Study variable	Item	Range	Mean (*SD*)
Gender	What is your gender? (Male or Female)	1–2	1.48 (0.50)
Age	How old are you?	16–20	17.30 (1.12)
Father’s education	No education to university education	1–5	4.40 (0.88)
Mother’s education	No education to university education	1–5	4.58 (0.88)
**5Cs of PYD**			
Character (8 items; α = 0.93)	Sample item: Doing what I believe is right even if my friends make fun of me.	1–5	3.94 (0.69)^a^
Confidence (6 items; α = 0.86)	Sample item: I really like the way I look.	1–5	3.73 (0.97)^a^
Connection (8 items; α = 0.89)	Sample item: I am a useful and important member of my family.	1–5	3.82 (0.77)^a^
Caring (6 items; α = 0.85)	Sample item: It bothers me when bad things happen to any person.	1–5	4.29 (0.78)^a^
Competence (6 items; α = 0.88)	Sample item: I have a lot of friends.	1–5	3.65 (0.86)^a^
Attitudes toward pollution	Pollution has increased	1–5	3.93 (1.17)
3 items (α = 0.41)	Pollution has dangers	1–5	3.49 (1.42)
	Pollution over growth	1–5	3.19 (1.22)
Environmental conservation	Conservation behavior 1	1–4	2.15 (0.88) ^a^
Behaviors	Conservation behavior 2	1–4	
3 items (α = 0.81)	Conservation behavior 3	1–4	
Behavioral intention (1 item)	Conservation intention	1–5	3.40 (1.52)
Environmental responsibility			
	Personal	1–4	2.18 (0.86)
	Consumers	1–5	3.95 (1.31)
Government	Government item 1	1–5	3.58 (1.25) ^a^
3 items (α = 0.90)	Government item 2	1–5	
	Government item 3	1–5	

*^a^Mean score; PYD, Positive Youth Development; α, Cronbach’s alpha; SD, Standard Deviation.*

Furthermore, to investigate the associations between the demographic variables, the 5Cs and the environmental variables, a correlation analysis was first conducted and then followed by a series of hierarchical multiple regression analysis. The regression analyses were used to test the hypothesis that higher scores on the 5Cs would be associated with higher scores on attitudes, behavior, and responsibility for the environment. Age, gender and parents’ educational background were included in the analysis as control variables, in step 1, as previous studies have shown that they tend to influence the individual’s experience of PYD indicators (Wiium et al., 2019). The 5Cs were added as independent variables in step 2 of the analysis. The dependent variables were attitudes, behavior and responsibility perception for the environment in separate regression models. G*Power 3 (Faul et al., 2009) was used to conduct a power analysis to determine the sample size that will allow for the assessment of meaningful associations and the detection of effect sizes (small, medium, or large). Using a two-tailed test with the nine independent variables (i.e., four demographic variables and the 5Cs), and an alpha value of 0.05, the results indicated that with a power of 0.80, sample sizes of 776, 98, and 39 were needed to detect effect sizes of 0.02 (small), 0.15 (medium), and 0.35 (large). Thus, with our sample size of 220, we were able to detect medium to large effect sizes in the regression analysis.

## Results

### Descriptive Analysis

Results from the frequency and descriptive analysis are presented in Table 1. For the 5Cs, participants reported having the greatest experience of *Caring*, with a mean score of 4.29 (*SD* = 0.78), followed by *Character* (*M* = 3.94, *SD* = 0.69), *Connection* (*M* = 3.82, *SD* = 0.77), *Confidence* (*M* = 3.73, *SD* = 0.97), and *Competence* (*M* = 3.65, *SD* = 0.86), all on a scale from 1 to 5. On questions about attitudes toward pollution, most participants reported that pollution has increased in Norway over the last 10 years (*M* = 3.93, *SD* = 1.17), while they least agreed that Norway needs growth to survive and that this will lead to an increase in pollution (*M* = 3.19, *SD* = 1.22). The sample also appeared to report higher conservation intention (*M* = 3.40, *SD* = 1.52) than actual conservation behavior (*M* = 2.15, *SD* = 0.88). When asked who was responsible for the environment, participants reported that they felt the consumer was most responsible (*M* = 3.95, *SD* = 1.31), followed by the government (*M* = 3.58, *SD* = 1.25), and finally, personally (*M* = 2.18, *SD* = 0.86) (Table 1).

### Correlation Analysis

Weak to medium correlations were found between “Pollution has increased” and three of the Cs: *Character* (*r* = 0.23, *p* < 0.01), *Competence* (*r* = 0.19, *p* < 0.01), and *Caring* (*r* = 0.30, *p* < 0.01). There was no significant correlation between “Pollution has dangers” and any of the Cs (Table 2). A weak, negative correlation was found between “Pollution over growth” and *Competence* (*r* = −0.18, *p* < 0.05) suggesting that higher levels of perceived competence were associated with lower scores on “Pollution over growth.” Conservation behavior was weakly, but significantly correlated with *Connection* (*r* = 0.21, *p* < 0.01), *Caring* (*r* = 0.18, *p* < 0.05), and *Competence* (*r* = 0.16, *p* < 0.05). Conservation behavior also had medium correlation with *Character* (*r* = 0.36, *p* < 0.01). Thus, conservation behavior correlated positively with all 5Cs, except for *Confidence*. Conservation intention was positively but weakly correlated with *Character* (*r* = 0.28, *p* < 0.01) and *Caring* (*r* = 0.26, *p* < 0.01) (Table 2).

**TABLE 2:** Correlations among demographics, the 5Cs of positive youth development and environmental concern variables.

Study variables	1.	2.	3.	4.	5.	6.	7.	8.	9.	10.	11.	12.	13.	14.	15.	16.
1. Gender	−															
2. Age	–0.02	−														
3. Father’s education	–0.12	−0.20**	−													
4. Mother’s education	–0.10	−0.17*	0.38**	−												
5. Character^a^	0.07	–0.06	0.08	0.14	−											
6. Confidence^a^	−0.18*	–0.12	0.15	0.19*	0.65**	−										
7. Connection^a^	–0.02	−0.15*	0.15*	0.16*	0.65**	0.68**	−									
8. Caring^a^	0.28**	–0.05	–0.03	0.11	0.66**	0.33**	0.48**	−								
9. Competence^a^	−0.21**	–0.14	0.20*	0.27**	0.53**	0.78**	0.72**	0.35**	−							
10. Pollution has increased	0.14	0.07	0.08	0.04	0.23**	0.11	0.14	0.30**	0.19**	−						
11. Pollution has dangers	0.08	0.11	–0.07	–0.09	–0.05	–0.10	–0.09	–0.03	–0.12	0.06	−					
12. Pollution over growth	0.08	0.23**	–0.10	–0.08	–0.08	–0.13	–0.09	–0.07	−0.18*	–0.08	0.52**	−				
13. Conservation^a^ behavior	0.12	0.14	0.01	–0.12	0.36**	0.14	0.21**	0.18*	0.16*	0.24**	–0.07	–0.13	-			
14. Conservation intention	0.22**	–0.11	0.02	0.04	0.28**	0.06	0.10	0.26**	0.07	0.27**	0.02	–0.03	0.46**	-		
15. Environmental responsibility: personal	0.04	0.09	–0.04	0.10	0.42**	0.26**	0.29**	0.21**	0.27**	0.19**	–0.11	–0.08	0.71**	0.46**	-	
16. Environmental responsibility: consumers	0.21**	–0.02	0.04	0.02	0.34**	0.13	0.16*	0.38**	0.12	0.57**	0.04	–0.04	0.29**	0.44**	0.36**	-
17. Environmental^a^ responsibility: government	0.22**	–0.08	0.15*	0.08	0.45**	0.24**	0.28**	0.41**	0.28**	0.61**	0.10	–0.03	0.40**	0.44**	0.46**	0.68**

^a^Mean score; * p < 0.05; ** p < 0.01.

Personal responsibility for the environment had weak to medium correlation with *Character* (*r* = 0.42, *p* < 0.01), *Confidence* (*r* = 0.26, *p* < 0.01), *Connection* (*r* = 0.29, *p* < 0.01), *Caring* (*r* = 0.21, *p* < 0.01), and *Competence* (*r* = 0.27, *p* < 0.01). Consumer responsibility for the environment was found to be weakly and positively correlated with *Connection* (*r* = 0.16, *p* = 0.05), and moderately correlated with *Character* (*r* = 0.34, *p* < 0.01) and *Caring* (*r* = 0.38, *p* < 0.01). Government responsibility for the environment was positively but weakly correlated with *Confidence* (*r* = 0.24, *p* < 0.01), *Connection* (*r* = 0.28, *p* < 0.01), and *Competence* (*r* = 0.28, *p* < 0.01). The analysis also showed that government responsibility for the environment was moderately correlated with *Character* (*r* = 0.45, *p* < 0.01) and *Caring* (*r* = 0.41, *p* < 0.01) (Table 2). Thus, the correlations between the 5Cs and the environmental variables were mostly weak (0.10–0.29), and moderate (0.30–0.49), rather than strong (0.50–1.00) (Cohen, 1988). As for the correlations between the demographics, the 5Cs and the environmental variables, they were only weak with the highest being between gender and Caring (*r* = 0.28, *p* < 0.01), where girls were more likely to report the PYD indicator than boys.

### Hierarchical Regression Analysis

Eight hierarchical regression analyses were conducted with the environmental variables as dependent variables, three analyses for attitudes toward environment, two for conservation behavior and intention, and the remaining three for environmental responsibility. The estimation of variance inflation factor (VIF) of the 5Cs as predictors, did not reveal any instance of multicollinearity as the VIF values were between 1.93 and 3.55. In addition, an analysis of standard residuals did not show any outliers (Standardized Residual Minimum = −1.99, Standardized Residual Maximum = 2.19). In Tables 3A–C, results from step 2 of the analysis, where demographic variables were controlled for are presented. We refer to the adjusted *R*^2^ when we report the explained variance. For “Pollution has increased” (i.e., one dimension of attitudes toward environment), *Competence* (β = 0.31, *p* < 0.05) and *Caring* (β = 0.23, *p* < 0.05) were found to be significant predictors. The 5Cs explained 7% of the total variance (Table 3A), after the initial 2% explanation by the demographic variables in step 1. For “Pollution has dangers” (i.e., a second dimension of attitudes toward environment), none of the 5Cs were significant predictors of the environmental variable after controlling for age, gender, and parental education. The 5Cs explained less than 1% of the total variance in the variable. Similarly, for “Pollution over growth” (i.e., the third dimension of attitudes toward environment), the 5Cs only explained 2% of the variance in the variable and did not show any significant association with the environmental variable (Table 3B).

**TABLE 3A:** The 5Cs of positive youth development and attitudes toward environment: A hierarchical regression analysis.

	Attitudes toward environment																	

	**Pollution has increased**						**Pollution has dangers**						**Pollution over growth**					
***Demographics and predictors***	***B***	***95% CI***	***SE of B***	**β**	***p***	***R* ^2^**	***B***	***95% CI***	***SE of B***	**β**	***p***	***R* ^2^**	***B***	***95% CI***	***SE of B***	**β**	***p***	***R* ^2^**
Constant	–1.00	−4.44–2.44	1.74		0.57	0.09	2.24	−2.20–0.6.67	2.25		0.32	0.03	–0.09	−3.82–3.64	1.89		0.96	0.03
Gender	0.28	−0.11–0.67	0.20	0.12	0.16		0.18	−0.32–0.68	0.25	0.06	0.48		0.16	−0.27–0.58	0.21	0.06	0.47	
Age	0.12	−0.04–0.28	0.08	0.11	0.16		0.11	−0.09–0.32	0.10	0.09	0.28		0.23	0.06–0.40	0.09	*0.21*	*0.01*	
Father’s education	0.15	−0.07–0.37	0.11	0.11	0.18		–0.03	−0.31–0.25	0.14	–0.02	0.82		–0.06	−0.29–0.18	0.12	–0.04	0.63	
Mother’s education	–0.05	−0.27–0.17	0.11	–0.04	0.65		–0.05	−0.34–0.23	0.14	–0.03	0.70		0.02	−0.22–0.26	0.12	0.02	0.86	
Character	0.17	−0.26–0.60	0.22	0.10	0.44		0.05	−0.50–0.61	0.28	0.03	0.86		–0.00	−0.47–0.47	0.24	0.00	0.99	
Confidence	–0.16	−0.50–0.70	0.17	–0.14	0.33		–0.01	−0.44–0.42	0.22	–0.01	0.96		–0.00	−0.36–0.36	0.18	–0.00	0.99	
Connection	–0.24	−0.61–0.13	0.19	–0.16	0.21		–0.04	−0.52–0.43	0.24	–0.02	0.86		0.17	−0.23–0.57	0.20	0.11	0.40	
Caring	0.34	0.02–0.66	0.16	*0.23*	*0.04*		–0.04	−0.45–0.38	0.21	–0.02	0.87		–0.11	−0.45–0.24	0.18	–0.07	0.55	
Competence	0.42	0.05–0.78	0.19	0.31	*0.03*		–0.11	−0.58–0.36	0.24	–0.07	0.65		–0.26	−0.66–0.14	0.20	–0.18	0.20	

*B, Unstandardized coefficient; SE, Standard error; β, Standardized coefficient; p, Level of significance; R^2^, Adjusted.*

**TABLE 3B:** The 5Cs of positive youth development and conservation behavior and intention: A hierarchical regression analysis.

	Environmental behavior and intention											

	**Conservation behavior**						**Conservation intention**					
***Demographics and predictors***	***B***	***95% CI***	***SE of B***	**β**	***p***	***R* ^2^**	***B***	***95% CI***	***SE of B***	**β**	***p***	***R* ^2^**
Constant	–1.48	−3.90–0.93	1.22		0.23	0.19	2.70	−1.70–7.11	2.23		0.23	0.10
Gender	0.22	−0.06–0.49	0.14	0.12	0.12		0.49	−0.01–0.99	0.25	*0.16*	*0.05*	
Age	0.12	0.00–0.23	0.06	*0.15*	*0.04*		–0.14	−0.34–0.06	0.10	–0.10	0.18	
Father’s education	0.06	−0.09–0.22	0.08	0.06	0.42		0.02	−0.26–0.30	0.14	0.01	0.88	
Mother’s education	–0.17	−0.33—0.02	0.08	*–0.17*	*0.03*		0.01	−0.28–0.29	0.14	0.00	0.96	
Character	0.73	0.42–1.03	0.15	*0.58*	*0.00*		0.82	0.27–1.37	0.28	*0.38*	*0.00*	
Confidence	–0.26	−0.49—0.03	0.12	*–0.29*	*0.03*		–0.28	−0.70–0.15	0.21	–0.18	0.20	
Connection	–0.00	−0.26–0.26	0.13	–0.00	0.99		–0.26	−0.74–0.22	0.24	–0.13	0.28	
Caring	–0.21	−0.43–0.02	0.11	0.19	0.07		0.10	−0.32–0.51	0.21	0.05	0.65	
Competence	0.23	−0.03–0.49	0.13	0.23	0.08		0.18	−0.29–0.65	0.24	0.10	0.45	

*B, Unstandardized coefficient; SE, Standard error; β, Standardized coefficient; p, Level of significance; R^2^, Adjusted.*

**TABLE 3C:** The 5Cs of positive youth development and environmental responsibility: A hierarchical regression analysis.

	Environmental responsibility																	

	**Personal**						**Consumer**						**Government**					
***Demographics and predictors***	***B***	***95% CI***	***SE of B***	**β**	***p***	***R* ^2^**	***B***	***95% CI***	***SE of B***	**β**	***p***	***R* ^2^**	***B***	***95% CI***	***SE of B***	**β**	***p***	***R* ^2^**
Constant	–0.55	−2.90–0.18	1.19		0.65	0.20	0.33	−3.36–4.03	1.87		0.86	0.14	–0.58	−3.89–2.73	1.68		0.73	0.25
Gender	0.08	−0.18–0.35	0.13	0.05	0.54		0.35	−0.07–0.77	0.21	0.13	0.10		0.48	0.10–0.85	0.19	*0.19*	*0.01*	
Age	0.07	−0.04–0.18	0.06	0.10	0.19		0.00	−0.17–0.17	0.09	0.00	0.97		–0.04	−0.19–0.12	0.08	–0.03	0.66	
Father’s education	–0.03	−0.18–0.12	0.08	–0.03	0.73		0.11	−0.12–0.34	0.12	0.08	0.35		0.22	0.01–0.43	0.11	*0.16*	*0.04*	
Mother’s education	–0.16	−0.31—0.01	0.08	*–0.16*	*0.04*		–0.06	−0.30–0.17	0.12	–0.04	0.59		–0.09	−0.30–0.12	0.11	–0.06	0.41	
Character	0.67	0.38–0.96	0.15	*0.54*	*0.00*		0.52	0.06–0.98	0.23	*0.28*	*0.03*		0.70	0.29–1.11	0.21	*0.39*	*0.00*	
Confidence	–0.15	−0.37–0.08	0.11	–0.17	0.20		–0.07	−0.42–0.29	0.18	–0.05	0.72		–0.18	−0.50–0.14	0.16	–0.14	0.27	
Connection	0.02	−0.24–0.27	0.13	0.01	0.91		–0.22	−0.62–0.18	0.20	–0.13	0.27		–0.24	−0.59–0.12	0.18	–0.15	0.19	
Caring	–0.19	−0.41–0.03	0.11	–0.17	0.09		0.38	0.03–0.72	0.17	*0.23*	*0.03*		0.22	−0.09–0.53	0.16	0.14	0.17	
Competence	23	−0.02–0.48	0.13	0.23	0.07		0.07	−0.32–0.47	0.20	0.05	0.71		0.37	0.02–0.72	0.18	*0.26*	*0.04*	

*B, Unstandardized coefficient; SE, Standard error; β, Standardized coefficient; p, Level of significance; R^2^, Adjusted.*

For environmental conservation behavior and intention, the regression analysis for conservation behavior revealed that after controlling for the demographic variables, *Character* (β = 0.58, *p* < 0.001), and *Confidence* (β = −0.29, *p* < 0.05) were found to be significant predictors of the behavior, although the association with *Confidence* was negative. The 5Cs explained 16% of the variance in conservation behavior. For conservation intention, the findings showed that only *Character* (β = 0.38, *p* < 0.05) was observed to be a significant predictor. The 5Cs explained 6% of the variance in conservation intention (Table 3C).

Hierarchical multiple regression analysis was also used to predict environmental responsibility (Table 3C). For personal responsibility, only *Character* was found to be a significant predictor, after controlling for the demographic factors (β = 0.54, *p* < 0.001). The 5Cs explained 20% of the variance in personal responsibility for the environment. For consumer responsibility, the analysis revealed that *Character* (β = 0.28, *p* < 0.05) and *Caring* (β = 0.23, *p* < 0.05) were statistically significantly related to the outcome variable. The 5Cs explained 11% of the variance in consumer responsibility for the environment. As for government responsibility to the environment, the analysis showed that *Character* (β = 0.39, *p* < 0.05) and *Competence* (β = 0.26, *p* < 0.05) were significant predictors. The 5Cs explained 19% of the variance in government responsibility for the environment. Moreover, several of the demographic variables, such as gender, age, and mother’s education were found to be significantly associated with the environmental variables (Tables 3A–C).

## Discussion

The current findings suggest that Norwegian youth’s experience of the 5Cs had significant influence on their attitudes toward the environment (i.e., “Pollution has increased”), environmental conservation behavior and intention, and environmental responsibility (personal, consumer, and government). When the contribution was significant in the examined models, the 5Cs explained between 6% of the variance in conservation intention and 20% of the variance in personal responsibility for the environment. Character predicted several of the eight environmental factors (five), followed by Caring (two) and Competence (two), and finally Confidence (one). Connection did not have any significant association with the environmental factors in regression analysis, and the analysis as well did not show any significant associations of the 5Cs with “Pollution has dangers” and “Pollution over growth.” Thus, the positive findings partially support the study’s hypothesis that higher scores on the 5Cs will be associated with higher scores on environmental factors.

Character’s prediction of attitudes toward increased pollution, environmental conservation behavior and intention, as well as perception of personal, consumer and government responsibility for the environment is in line with findings from earlier research, such as van der Werff et al. (2013) and Kabir and Wiium’s (2021) study. van der Werff et al. (2013) assert that motivation to act environmentally friendly can be explained as commitment-based inner motivation and compared this type of motivation to a personal norm. The authors found results that may indicate that personal norm was likely to mediate the relationship between eco-friendly identity and intentions to use renewable energy. Within the PYD framework, Character reflects good morals and integrity. Assuming that Character can be equated to the concept of personal norms, findings from van der Werff et al. (2013) and others, such as Balunde et al. (2020) could underscore the influence of Character on environmental concern in the present study. Moreover, van der Werff et al. (2013) found that environmentally friendly identity might be positively associated with the personal norm of engaging in environmentally friendly behaviors. This finding can be linked to our findings on the associations of Character with conservation behavior and personal responsibility.

Furthermore, significant correlations were found between Confidence and personal as well as government responsibilities for the environment, although a negative association between the variable and conservation behavior was also observed. While the latter finding is contrary to our predictions, for the former, it is conceivable that the belief that one can do something for the environment can be translated to a personal sense of responsibility, while also expecting the government to do its part. Moreover, van der Werff et al. (2013) suggested that it may be possible to strengthen an individual’s environmentally friendly identity by reminding them of environmentally friendly behaviors they already perform. This earlier finding can be linked to our positive correlations of Competence with conservation behavior and personal environmental responsibility, although we do not find these significant associations in regression analysis when demographic factors are controlled. By reminding individuals of the usual actions they already do that are environmentally friendly, they may be motivated to continue the behavior. Similarly, the significant correlation between Caring and conservation behavior as well as personal responsibility to the environment (although the significant associations disappear in regression analysis) are supported by Metzger et al. (2018) who found that empathy and future orientation predicted almost all forms of social engagement, including environmental behavior. Thus, the correlations to some extent, build on earlier findings that there is an association between empathy and community engagement. Finally, Connection, which has been defined as the experience of positive relationships with people and institutions (Lerner, 2004), was only significantly associated with the environmental factors in correlation analysis. For the significant associations to disappear in regression analysis could indicate the mediating effect of the other Cs as the 5Cs, although distinct, have been found to correlate with each other. For the demographics, age and gender were mostly related to the environmental factors. That higher scores on positive attitudes toward the environment and conservation behavior were observed among older participants relative to their younger counterparts as well as the finding that females reported more conservation intention than males need to be further explored to ascertain the role of demographic factors in environmentally sustainable behaviors.

### Limitations

Despite the significant findings on the link between the 5Cs and the environmental factors, the present study presents some limitations. First, the attitudes toward pollution scale had a low Cronbach’s alpha and thus the items were treated separately rather than as a scale. Besides, in Norway, one cannot obtain a driver’s license until they are 18 years of age, unlike in the US where the age limit is 16 years. The mean age of participants in the present study was 17.30 years. Thus, environmental questions that focus on Norwegian youth’s use of public transport or bicycle rather than driving a car would not necessarily depict their own conservation behaviors. Whether the environmental factors are good enough to capture Norwegian youth’s attitudes, behavior and responsibility for the environment is a topic that needs to be explored further in future studies with qualitative methods. Besides, the single-item measures used in assessing attitudes toward pollution may not be valid or reliable enough. This is a limitation that needs to be addressed with more adequate measures in future research.

In addition, strong correlations were observed between some of the 5Cs, although the estimation of variance inflation factor did not indicate a case of multicollinearity. Theoretically, the five distinct components of PYD are related, and the strength of the relation may vary across samples. Future assessment of the components can investigate the extent to which the relations are due to the formulation of the measurement items or participants understanding of them.

Furthermore, although power analysis showed that our sample size was adequate to detect medium to large effect sizes, the sample was 220 high school students, who attended schools that were selected through convenience sampling. This will likely limit the generalizability of the findings to the youth population in Norway. Moreover, the cross-sectional design that was used does not allow for any causal interpretations of the findings. Future research with a larger and more representative sample and ideally, a longitudinal design is certainly needed to verify the links between the 5Cs and the environmental factors.

### Implications for Research, Policy, and Practice

The present study is the first to investigate the 5Cs of PYD and associations with environmental attitudes, behaviors and responsibilities in a Norwegian context, thus breaking the ground for more studies in the future, in Norway and other Scandinavian and European contexts. As the present study used a cross-sectional design, it can be interesting to investigate further the findings with longitudinal designs and more up-to-date environmental factors. For example, it may be of interest to investigate environmental concerns regarding issues that deal with rising sea levels, damage to the natural environment, meat consumption and Norway’s petroleum activities. The investigation of these different forms of youth contribution can eventually help to extent research on the sixth C of PYD (i.e., Contribution). Demographic factors and their role can also be explored in future research.

As mentioned in the introduction, young people have been an important target group for political parties in Norway that focus on the environment (Sørenes, 2019). The current findings suggest that experiencing the 5Cs can enable the youth to contribute actively to the environment. Thus, political actions that ensure positive development and opportunities for the youth may be empowering them to take personal responsibility for the environment.

Moreover, concerning implication for practice, youth initiatives that are set up to promote commitment to the climate and environment may benefit by creating a context that facilitate positive development and the 5Cs. With this, youth organizations working for the environment should not only focus on increasing knowledge, but also on promoting positive development.

## Conclusion

The experience of the 5Cs among youth in Norway was observed to be significantly related to several environmental factors regarding their attitudes, behavior, and responsibility perception. Character was found to be mostly associated with the environmental factors, followed by Caring and Competence and finally Confidence. Connection had no significant association with the environmental factors in multivariate analysis. The current study offers preliminary findings that suggest that promoting the experience of the 5Cs among youth can generally lead to contributions to the environment through their attitudes, behaviors and sense of responsibility. Thus, youth organizations working for the environment should not only focus on increasing knowledge, but also on promoting positive development. In addition, political actions that ensure a higher experience of the 5Cs among young people will be facilitating their contribution to sustainable development. However, there is a need for further research in the field and the examination of whether the findings can be replicated in a larger sample, using longitudinal studies and alternative environmental factors, not just in Norway, but also in other Scandinavian and European countries.

## Data Availability Statement

The raw data supporting the conclusions of this article will be made available by the authors, without undue reservation.

## Ethics Statement

The studies involving human participants were reviewed and approved by NSD–Norwegian Centre for Research Data (51708/3/IJJ). Written informed consent from the participants’ legal guardian/next of kin was not required to participate in this study in accordance with the national legislation and the institutional requirements.

## Author Contributions

MB was responsible for data collection, original draft writing, and revisions. NW was responsible for conceptualization, methodology, software, formal analysis, original draft writing, and revisions. Both authors read and approved the version for publication.

## Conflict of Interest

The authors declare that the research was conducted in the absence of any commercial or financial relationships that could be construed as a potential conflict of interest.

## Publisher’s Note

All claims expressed in this article are solely those of the authors and do not necessarily represent those of their affiliated organizations, or those of the publisher, the editors and the reviewers. Any product that may be evaluated in this article, or claim that may be made by its manufacturer, is not guaranteed or endorsed by the publisher.
